# “Maybe in a few years I'll be able to look at it”: a preliminary study of documentary issues in the Ukrainian refugee experience

**DOI:** 10.1007/s10502-022-09407-1

**Published:** 2022-12-23

**Authors:** Magdalena Wiśniewska-Drewniak, James Lowry, Nadiia Kravchenko

**Affiliations:** 1 Nicolaus Copernicus University in Toruń, Toruń, Poland; 2 City University of New York, New York, USA; 3 Taras Shevchenko National University of Kyiv, Kyiv, Ukraine

**Keywords:** Invasion of Ukraine, Russian Ukraine war, Refugees, Records, Documentation

## Abstract

Following the February 2022 invasion of Ukraine by Russia, millions of refugees have fled Ukraine for safety in neighbouring countries, including Poland. This movement of people has been facilitated by, and has produced, documentation that will have significant afterlives as evidence and memory. The records refugees have carried with them, the records they have made during flight, and the records created in their encounters with states and communities beyond their homeland, will be important in the prosecution of war crimes, the reconstruction of events, the reconstitution of communities and the protection of rights and entitlements. This article sets out the findings of a pilot study into the documentary experiences of Ukrainian refugees in Poland. Interviews conducted by and with Ukrainian refugees consider the removal of records, the documentation of the refugee experience, the documentary requirements of border crossings, and the informational requirements of life beyond the border. Although drawing on a limited study population, the research surfaces some significant issues related to the preservation of memory and culture, exclusionary and hostile government information systems, and research ethics. This article could help to inform archival solidarity with Ukraine; it underscores the need for trauma-informed archival research and practice; and finally suggests the need for a person-centered approach to this work.

## Introduction

On 24 February 2022, Russian forces invaded Ukraine, initiating the mass movement of Ukrainians into Poland and other neighbouring countries. This article outlines the results of a pilot study of documentary issues connected with the Ukrainian refugee experience. It is intended to help inform further research in archival studies, and information studies more broadly, that could address the informational, memorial and evidential implications of the invasion of Ukraine. The impetus for this research comes from the questions under discussion in the Ukrainian, Polish and international archival communities about how archivists and others should respond to the situation created by Russian aggression. The article outlines the context of the war in Ukraine, summarises the response of the archival community to date, details the impetus and formation of the pilot study, before setting out its findings and their implications. It thereby suggests some directions for further work.

Because this research concerns a contemporary situation, apart from our interview data, many of the sources used in this article are issued by international bodies, state authorities, humanitarian or civil society organisations, professional associations and the news media. The information in the article is current as at 18 July 2022. There is little scholarly work related directly to the topic of records, refugees and the Russian invasion of Ukraine, but our work is informed by the work of the Rights in Records in Displacement and Diaspora Network (RRDDN), a worldwide community of scholars, information and cultural professionals, educators, artists, activists, and those who have experiences of displacement, migration and diaspora, which seeks to promote the rights of individuals and peoples to create, keep, carry, access, use and share records, documentation and information technologies. The genesis of this work is in Gilliland (2017). The present research is also informed by work in community and diasporic archives, as outlined in the methodology, and by developments in archival theory in the areas of critical archival studies and trauma-informed archival practice, as reflected in our findings. In this article we do not make a central argument, attempt to integrate our data with existing literature or frameworks nor to contribute to theory-building. Instead, we present some preliminary findings which may inform current and future action in solidarity with Ukraine and in ameliorating the Ukrainian refugee experience internationally.

### Russia’s invasion of Ukraine in 2022

The history of the ongoing (as at July 2022) Russian-Ukrainian war dates back to at least 2014, when Russia illegally invaded the territory of the Crimean peninsula, which has been occupied by Russian forces ever since (Rogoża and Fischer 2014). The year 2022 brought another, broader and more dramatic, development related to this conflict. On 21 February 2022, the Kremlin recognised the independence of the Donetsk People's Republic and the Luhansk People's Republic—Russian-backed areas in eastern Ukraine that declared independence from Ukraine in May 2014 on the basis of internationally unrecognised referenda (Szumski 2015). Three days later, on 24 February 2022, Russian troops invaded Ukrainian territory under the pretext of its 'demilitarisation' and 'denazification'—emphasising the need to disarm Ukraine and to stop its military cooperation with the West (Żochowski and Nieczypor 2022). At the same time, the Russian official narrative questions Ukraine's right to independence, statehood and a distinct Ukrainian identity (Mankoff 2022). Belarus is also indirectly and unofficially involved in the conflict, among other things, making its territory available to Russian units to attack Ukraine: troops thus entered Ukraine from the territory of both Russia and Belarus (Kłysiński 2022).

Although the war between Russia and Ukraine, with the involvement of Belarus, may appear to be a local conflict, it is in fact an important event in the increasingly difficult relationship between Russia and Europe, and, more broadly, the North Atlantic Treaty Organisation (NATO) (Richter 2022).

However, Russia does not use the word 'war' in official communications. Instead, Russian and Russia-sympathising authorities and media use the phrase “special military operation” to conceal the aggressive and destructive character of Russia’s activity in Ukraine (Gorobets 2022). In this article we clearly express our view of Russian aggression by calling it a war, following in the footsteps of most of the world's countries and international organisations, e.g. the United Nations (UN 2022), the European Union (European Council 2022), and NATO (NATO 2022).

On 24 February 2022, Russia's invasion of Ukraine began in the early hours of the morning. The citizens of the democratically governed, independent state were awakened by the sounds of explosions. Many Ukrainians made the decision to leave their homes immediately; some only did so in the following days and weeks, touched not only by direct violence from Russian troops but also by a severe humanitarian crisis, including power shortages, lack of heating, the interruption of health care and social services, and in occupied areas, water and food scarcity (United Nations Office for the Coordination of Humanitarian Affairs n.d.; International Committee of the Red Cross n.d.).

The rules of general mobilisation, issued on 24 February 2022 by the President of Ukraine, V. Zelensky (2022), did not allow men aged 18 to 60 to leave the country, apart from those exempt under the general law about mobilisation of 1993. Among these exceptions were: persons performing service in bodies of state authorities other than the military; persons declared temporarily unfit for military service; men and women who have three or more dependent children under the age of 18; single parents; persons raising a disabled child and persons caring for a disabled adult; carers and foster parents. Among the persons excluded from the obligation to report for military service there is also a place for members of the academic community: students and doctoral students, as well as research and teaching staff employed in higher education institutions and scientific organisations (Law of Ukraine No. 44, article 23 1993). However, men in this category are also disallowed from leaving the country. As a result of these regulations, the vast majority of refugees, over 90%, are women and children.

It is important to mention at this point that although the article focuses on refugees leaving Ukraine, since our data concerns cases of women who have crossed the Ukrainian-Polish border, the movement of individuals and families within Ukraine is very intensive and results in a significant number of internally displaced persons. According to the UN Refugee Agency

Since 24 February 2022, one-third of Ukrainians have been forced from their homes. This is the largest human displacement crisis in the world today. Some 7 million people have been displaced internally within Ukraine and some 13 million people are estimated to be stranded in affected areas or unable to leave due to heightened security risks, destruction of bridges and roads, as well as lack of resources or information on where to find safety and accommodation (UNHCR n.d.a).

At the time we started working on this project (end of May 2022) the number of people who had left Ukraine since the start of the invasion was more than 6.6 million. Most of them crossed the border with Poland (over 3.5 million), Romania (972.000), Russia (945.000), and Hungary (650.000) (UNHCR n.d.b; n.d.c). Those who moved to Russia were largely deported there by the occupying Russian authorities in eastern Ukraine (Żochowski and Nieczypor 2022). As we finish writing this article in mid-July 2022, the figures look different as many Ukrainians have moved west. When it comes to the neighbouring countries, there are now more than 1.6 million Ukrainians in Russia. There are currently more than 80.000 Ukrainians in Moldova, Romania and Slovakia in each of these countries, more than 25.000 in Hungary, almost 11.000 in Belarus. More than 1.2 million of Ukrainians currently reside in Poland, although the number of border crossings from Ukraine is much higher (over 4.6 M).

Cross-border movement has not been unidirectional. The number of border crossings from Poland to Ukraine is also very high (over 2.5 M). According to the UNHCR.

Movements back to Ukraine may be pendular, and do not necessarily indicate sustainable returns as the situation across Ukraine remains highly volatile and unpredictable (UNHCR n.d.b).

As eligible Ukrainians moved into Poland and other neighbouring countries, large numbers of Ukrainian men resident in Poland, many of them construction workers, returned to defend Ukraine, to the extent that major construction projects in Poland were halted or delayed. Currently, there continues to be traffic across the border, as more refugees flee the advancing Russian forces, and Ukrainians continue to return to fight the invaders or otherwise support the war effort, seek out missing family members, check on abandoned properties, etc. At the time of writing, the Ukrainian member of our research team is in Kyiv, returning to her family.

Many Ukrainians who crossed the Polish-Ukrainian border have moved west, often visiting their friends or relatives who had already lived and worked in Western Europe before the war. Currently, the largest number of Ukrainians are registered in Germany (893.000), Czech Republic (372.000), Italy (145.000), Turkey (145.000), Spain (128.000), UK (95.000), France (92.000), Bulgaria (88.000), Austria (74.000), Netherlands (68.000), Lithuania (59.000), Switzerland (57.000), Belgium (50.000) (UNHCR n.d.b).^1^

The high number of refugees is partly attributable to the nature of the conflict, i.e. among other things, the fact that Russian troops target civilian infrastructure. Since 24 February 2022, the Office of the UN High Commissioner for Human Rights (OHCHR) recorded over 11.500 civilian casualties in Ukraine (over 5.000 killed, over 6.500 injured; including women and children). According to OHCHR:

“Most of the civilian casualties recorded were caused by the use of explosive weapons with wide area effects, including shelling from heavy artillery and multiple launch rocket systems, and missile and air strikes.

OHCHR believes that the actual figures are considerably higher, as the receipt of information from some locations where intense hostilities have been going on has been delayed and many reports are still pending corroboration” (United Nations Human Rights Office of the High Commissioner 2022).

As we write these words now, more than 140 days of Russian aggression against Ukraine have passed. Intense fighting continues mainly in eastern Ukraine, including around Kharkiv, and the city itself is regularly shelled by the Russians (this is where one of our interviewees is from). The Ukrainian army continues to receive support especially from NATO countries, including modern military equipment that soldiers need to be trained to use. A puppet pro-Russian administration operates in the eastern occupied territories (Nieczypor et al. 2022). Ukrainian officials are currently examining more than 15.000 possible war crimes. The US and Europe are also supporting an International Criminal Court investigation (Schifrin 2022). Recent shocking Russian attacks on civilian infrastructure include the bombing of a shopping centre in Kremenchuk on 27 June (20 killed, almost 60 injured) (BBC News 2022). On 1 July, Russian rockets hit the Serhiyivka resort in the Odessa region. It resulted in the deaths of at least 21 people (Odintsova 2022). On 9 July, in the evening, Russian troops carried out a rocket attack, which resulted in damage to a five-story residential building in Khasovy Yar (48 fatalities) (Censor.Net 2022). On 14 July, Russian missiles struck the city centre of Vinnytsia, destroying two community facilities, houses, a medical centre, cars and trams. Twenty-three people including three children were killed, 66 injured, 39 are still missing (Karazy 2022).

### International and local archival responses to the Russian invasion of Ukraine

On the day the invasion began, UNESCO called for respect for international law, including humanitarian law and laws pertaining to the protection of cultural heritage, and on the following day, the Blue Shield issued a statement calling for compliance with international law. Subsequently, various national Blue Shield committees have sent “financial assistance and shipments of conservation equipment” to Ukraine (Blue Shield International 2022). A UNESCO press release on 3 March 2022, following the UN General Assembly’s Resolution on Aggression in Ukraine, drew attention to the impacts of the war on cultural sites, such as the damage to the Babyn Yar Holocaust memorial, on education, mentioning the nationwide closure of schools and attacks on universities, and on freedom of information and expression, citing the Russian targeting of media infrastructure such as television towers. The same day, UNESCO reported working with Ukrainian heritage professionals to mark historic sites with the 1954 Hague Convention emblem (the “Blue Shield”), and working with the United Nations Institute for Training and Research to analyse satellite imagery of cultural sites.

Following an extraordinary meeting of the UNESCO Executive Board, the *Declaration on the Protection of Cultural Heritage in Ukraine* was issued on 18 March 2022, again calling for respect for international law and for Russia to prevent the pillage of cultural heritage. UNESCO’s Committee for the Protection of Cultural Heritage in the Event of Armed Conflict held an emergency meeting four days later, where *inter alia* they granted a preliminary USD $50.000 to in situ preservation and the evacuation of heritage materials (UNESCO 2022). On 29 March 2022, the Minister of Culture and Information Policy of Ukraine, Oleksandr Tkachenko, called on UNESCO to exclude Russia and for the 45th session of the World Heritage Committee, planned to be held in Russia, to be relocated. In late April the meeting was “indefinitely postponed” (Ludel 2022). In subsequent months, UNESCO has developed programmes to support journalists, artists and museum curators in Ukraine and in exile, and has continued to work with Ukrainian cultural heritage workers (UNESCO 2022).

The Galt Museum and Archives in Alberta, Canada, began collecting financial donations for the State Archival Service of Ukraine. The President of the International Council on Archives (ICA), David Fricker, encouraged ICA members to donate to this fund, stating “The elected officers and I believe that using an existing channel to financially support our Ukrainian colleagues is the most effective way to have a direct impact on rebuilding Ukrainian archival capacity.” (International Council on Archives 2022a) The ICA leadership was in frequent communication with the State Archivist of Ukraine and colleagues in neighbouring countries “to monitor this fast-moving situation and provide what assistance it can to colleagues on the ground and to safeguard at risk archives” (International Council on Archives 2022b). The ICA issued a statement of solidarity with Ukraine and called on Russia to respect the 1954 Convention for the Protection of Cultural Property in the Event of Armed Conflict. Statements have also been issued by the Society of American Archivists (2022), the Australian Society of Archivists and the Records and Information Management Professionals Australasia (2022), and the Digital Preservation Coalition (2022), and the Association of Canadian Archivists has raised funds for Ukrainian archives (Association of Canadian Archivists 2022).

On 10 March 2022 an extraordinary meeting of the Executive Board of the ICA voted to suspend relations with the four Russian and Belarussian state archival institutions that were ICA members, a resolution subsequently opposed by Chinese representatives at the ICA Executive Board meeting in May 2022: the suspension remains in force at the time of writing.

The ICA resolution also drew criticism from prominent Russian archivist Natasha Khramtsovsky, who condemned the move in a now deleted blog post, in which she rightly noted the ICA’s silence on past US foreign invasions, though failing to condemn Russia’s war crimes. As a result, her interview in a forthcoming issue of “Archives and Records” was pulled, one of a range of responses from the UK and Ireland’s Archives and Records Association that also included a statement of solidarity (28 February), promotion of CILIP’s statement (for signature) and ongoing communication with Ukrainian and European colleagues. According to the website of the Archives and Records Association (2022):

“The Estonian state archivist reports that on the initiative of the National Archives of Poland and three Baltic States… have also been in direct contact with the headquarters of the General Administration of Archives of Ukraine in Kyiv in recent days. As a result of these contacts, it is known that, given the current situation on the ground, Ukrainian colleagues do not consider it is possible to provide them with any assistance from outside Ukraine other than the moral support for their state and the archives at present. The evacuation of large numbers of records across borders in the current context of active war is unthinkable. The Estonian state archivist has been in direct contact with the head of the Ukrainian Institute of National Remembrance, who confirmed exactly the same - it is currently not possible to provide any international physical assistance. Nonetheless, the heads of the Lithuanian, Latvian and Estonian archives have discussed and mapped out the possibilities in the countries closest and friendly to Ukraine, should it be possible and necessary to provide evacuation assistance and find storage facilities for evacuated collections.”

Over time, Polish archives have become involved in responding to the needs of Ukrainian archives in, among other things, providing fire-fighting equipment and equipment for the protection of archival materials on site (Archiwa Państwowe on Facebook 2022b). At the same time, Poland’s General Directorate of State Archives in its statement in March said:

We are constantly monitoring the situation and conducting a coordinated relief effort. We are ready to provide all necessary support if it is requested by the Ukrainian archives service. At the same time, we would like to point out that due to security reasons, we will officially present only partial information on this subject (Archiwa Państwowe 2022a).

In early March, the Polish state archives also announced an aid campaign for Ukrainian archivists and their families who had fled to Poland in the aftermath of the war. Two contact points were opened in the archives closest to the Ukrainian border (Przemyśl and Chełm), offering support in temporary accommodation and financial support for necessities (Archiwa Państwowe on Facebook 2022a).

Polish state archives have also engaged in symbolic support for the Ukrainian cause. In May, an exhibition installed in the city space of Piotrków Trybunalski and organised by the local archive, entitled 'Ukrainian traces in the Piotrków Trybunalski land. Documents from the resources of the State Archive in Piotrków Trybunalski’ (Archiwa Państwowe 2022b).^2^

A Polish-Ukrainian archival project 'Mom, I see war' is also currently underway. On 19 July 2022, an exhibition presenting children's images of war is to be opened. It will comprise Polish children's drawings from 1946, stored in the state archives, which are a record of their experiences during World War II and the German occupation of 1939–1945, and contemporary drawings by Ukrainian children depicting the ongoing war, collected on the "Mom I see war" portal (https://momiseewar.com/). The exhibition will be presented in 25 Polish cities (Archiwa Państwowe 2022c).

The research and documentation project '24.02.2022, 5am: Testimonies of War’ was initiated by the Centre for Urban History of Central and Eastern Europe from Lviv (Ukraine), which is recording interviews in Ukraine. The project was also joined by partners from Poland (Institute of Philosophy and Sociology of the Polish Academy of Sciences, Polish Oral History Association), Scotland (University of St Andrews) and Luxembourg (Centre of Contemporary and Digital History). Interviews are only conducted with adults, with a stable financial situation, who have been in Poland for at least four weeks. The interviewee can choose the language of the interview and stay anonymous (Projekt dokumentacyjny "Świadectwa wojny" on Facebook 2022).

The previous phase of the Russian—Ukrainian conflict (the annexation of Crimea in 2014 and the establishment of the so-called “people's republics” in Donetsk and Luhansk) has already prompted oral history projects. In 2015, the publishing project 'Oral History of the Russian-Ukrainian War' co-created by Ukraine's Institute of National Memory, Zaporizhia National University and the Zaporizhia Regional State Administration was launched. The project publishes excerpts from oral history interviews with participants in the war (e.g. military chaplains, women soldiers, volunteers, displaced persons) (Ukrainian Institute of National Memory n.d.). We know from brief media reports that the Institute of National Memory is working on another project to record witnesses to a war that is currently taking place (Farago 2022).

At the beginning of March 2022, i.e. about a week after the start of the war in Ukraine and the first waves of refugee migration to Poland, Archives of Aid (Polish: Archiwum Pomocy) was established in Wrocław. The institutions co-creating the archive are the History Centre “Zajezdnia” (a cultural institution jointly run by the Ministry of Culture and National Heritage and the Municipality of Wrocław), the Historical Institute of the University of Wrocław and the Paweł Włodkowic Institute (an NGO, independent research and educational centre). The Department of History at Adam Mickiewicz University has also become involved in the archive's activities (Adam Mickiewicz University 2022). The aim of the archives is to document aid actions organised in Poland for Ukrainians and solidarity between nations. The creators of the archives say:

“Today's solidarity between Poles and Ukrainians will shape our mutual relations for decades. It is a fundamental event in our common history. Let us not allow it to remain undocumented” (History Centre ‘Zajezdnia’ 2022).^3^

The creators encourage people to send to the archive via email, e.g. digital photographs and videos from mobile phones, photos of posters, banners, wall inscriptions, social media posts on aid, solidarity and support, online articles. The Archive also encourages people active in aid committees and organisations involved in helping Ukrainians to preserve electronic (including emails and instant messaging discussions) and traditional documentation and possibly transfer them to the Aid Archive (in digital or paper) (History Centre ‘Zajezdnia’ 2022). At the same time, it should be noted that there is no information on the transfer of rights to the submitted materials, very little information on archival description (description prepared by donors) or required or suggested file formats, as well as information on who will own these documents and how they will be used.

Aside from institutional responses, networks of individual archivists have organised to support the survival of Ukrainian materials. One prominent example is Saving Ukrainian Cultural Heritage Online (SUCHO), which is an international network of over 1.300 individuals working to archive Ukrainian web content, and increasingly the digital surrogates of Ukrainian archives. To date, SUCHO has archived over 5.000 websites, many of which are now offline as a result of Russian damage to infrastructure and online attacks. SUCHO is now moving into a new phase of work, “coordinat[ing] aid shipments of digitization hardware, exhibit[ing] Ukrainian culture online and organis[ing] training for Ukrainian cultural workers in digitization methods” (Saving Ukrainian Cultural Heritage Online n.d.).

While it is outside of scope for us to provide an overview of the many memory initiatives being led from outside the archival profession, it is worthwhile noting that public and especially digital memorials have been and are being created by the private sector (for example, the Victims of Russia’s war against Ukraine memorial created by the Abo Media Agency, https://www.victims.memorial/) and the non-profit sector (for example, the Rinat Akhmetov Foundation’s Museum of Civilian Voices, which was a response to the 2014 invasion and continues recording civilian accounts of the war in 2022, https://civilvoicesmuseum.org/en). Historians are also organising to document the war, with new training activities around interview-based research (Ukraina Moderna 2022).

While this overview of the international archival community’s response to the invasion of Ukraine does not detail all of the statements and initiatives of official entities, associations and individuals, it provides a broad overview as context for our study, which we hope will offer archivists and archival organisations useful preliminary insights into the records issues created by the movement of refugees as a result of Russian aggression.

### Preliminary research: method and scope

This research is grounded in Magdalena Wiśniewska-Drewniak’s ongoing research agenda focused on community archives in Poland. Between 2016 and 2019, she conducted a study funded by the National Science Centre in Kraków aimed at describing and understanding eight different community archives contemporarily operating in Poland. As a result of that work, the first Polish scientific monograph on community archives was published, entitled “Inaczej to zniknie. Archiwa społeczne w Polsce – wielokrotne stadium przypadku [Otherwise it disappears. Community archives in Poland – multiple case study]” (Wiśniewska-Drewniak 2019). Her recent focus on personal documentation practices, the social roles of archives and documentation and affectivity in the context of archival science, as well as the creation and use of Polish diaspora archives, was also significant in the identification of the need to consider the archival issues generated by the sudden influx of refugees from Ukraine to Poland. Recognising the need to understand the documentary practices of refugees, and states in respect of refugees, particularly in the specific context of Ukrainians in Poland, Magdalena Wiśniewska-Drewniak initiated local research.

Like many other universities across Poland, Nicolaus Copernicus University, located in the UNESCO World Heritage listed city of Toruń, redirected its resources to support Ukrainian refugees, particularly academics who were exempt from the ban on mobility. In mid-March 2022, the university created the 'Grants to Start' program to enable the hiring of scholars from Ukraine who wish to conduct research in collaboration with academics from Poland (Nicolaus Copernicus University 2022). However, even before this formal programme started, the Faculty of History at Nicolaus Copernicus University had used departmental funds to hire three Ukrainian colleagues on a short-term, emergency basis. Through this arrangement, Magdalena Wiśniewska-Drewniak was connected with Nadiia Kravchenko. She is a historian by training, currently preparing her PhD dissertation on the history of hunting in the sixteenth and seventeenth centuries. Nadiia Kravchenko’s positionality and lived experience as a Ukrainian refugee informed the design and execution of the research project.

One of the initial priorities was to interview Ukrainian war refugees residing in Poland about the role of documentation and archives in the process of fleeing their country and experiencing the war. Magdalena Wiśniewska-Drewniak and Nadiia Kravchenko jointly developed an interview protocol, informed by Nadiia Kravchenko's experience of escaping from Ukraine to Poland. The scenario consisted of 14 questions which concerned: information about the interviewee (name, age, profession, place of origin); experience of the first day of the war; ways of documenting the war and using this documentation; experience of crossing the Ukrainian-Polish border and the official side of this process (especially in the context of created and required documentation); and applying for the Polish ID number (PESEL), which allows one to access Polish government services.

Interviewees were identified through networks within NCU, there being no systematic way of communicating with a possible study population at that time. Three interviewees were identified: two women employed as guests at the NCU Faculty of History (Interviewee no. 2 and 3). The third interview was conducted with a woman who had personal connections with a NCU historian and Nadiia Kravchenko’s host (Interviewee no. 1). Although this is a very small study population, the interviews were undertaken to test the suitability of the interview protocol and to elicit some preliminary information that could inform the research design of a fuller study. This article reports only on the preliminary interviews, and does so to help to inform archival organisations and scholars as they seek to support the Ukrainian people, the survival of Ukrainian culture and evidence of Russian aggression.

Interviews were conducted in the second half of April 2022 (20 April, 25 April, 29 April). One took place in a coffee shop, one in a university library, one in the interviewee’s office at NCU. The interviews were recorded in audio format on a digital voice recorder with the consent of both parties.

As is usual in the humanities in Poland, the NCU Faculty of History does not have any type of institutional review board process, as faculty (with the exception of Magdalena Wiśniewska-Drewniak) do not normally conduct research involving human subjects that requires ethics approval. There is no similar body at university level, as this is dealt with by departmental committees in those departments that conduct research that requires a particular focus on ethical issues. Consequently, the project has not received formal ethics committee approval. In the absence of such a structure, in its design and conduct, the research project employed the Code of Ethics for Sociologists of the Polish Sociological Association, which, although designed for a different academic discipline, is well suited to the aims and scope of the project and the nature of the relationships between the researchers and participants.

Special attention was paid to the following principles:

- To endeavour to protect the interests of individuals and communities involved in or affected by the study;
- To take into account the consequences of the research project or its misuse for the participants or other interested parties;
- To strive to protect the rights of the participants, their interests, sensitivities, and privacy;
- To make every effort to ensure that participation in research is based on the voluntary and informed consent of the participants, both as to the purpose and conduct of the study and as to the uses to which it may be put;
- To undertake to verify that the study will not be discomforting to the participants and to try to find ways to minimize or mitigate discomfort that the participants may experience as a result. (Polish Sociological Association 2012)

All interviewees signed a consent form. The consent form contained: information about the interviewee and the interviewer, date of the interview, name of the project and project leader (with contact details). This was followed by a section about giving permission to use the interview for purposes related to the implementation of the project and other research and educational activities; to archive the interview and its transcript in the archives of the Department of Archival Science and Records Management at the Nicolaus Copernicus University in Toruń or in another research data repository selected by the project leader; to publish excerpts from the interview in scientific, educational and popularising publications (also after editing or translation), respecting the personal rights of the interviewee. Another part of the document concerned the anonymisation of the interview. The interviewee was able to decide for herself whether to ask for the interview to be anonymised (and to propose how she should be quoted, e.g. name, pseudonym), or whether she agreed to be given her full name. All interviewees chose the first option. According to their wishes, throughout the article they are referred to as Interviewee no. 1, 2, and 3. The document also contained a space where the research participants could add their objections to the conditions proposed in the document. The document was translated into Ukrainian by Nadiia Kravchenko to ensure that the research participants understood the conditions for handling the data collected during the project.

The personal and professional profiles of the interviewees is relevant to the analysis of the interview data in the following section, so it is necessary to provide a summary of the profile of each interviewee.

Interviewee no. 1, 27, is a landscape designer. She lives in Poland. She was in Lviv when the war started because she was visiting her parents. At 5am that day, her husband called saying: “pack soon, let your father take you to the border. The war has begun. Please forgive me for letting you go”. She crossed the border on the first day of the war, on foot, with a small child (under a year old).

Interviewee no. 2, 40, is a historian by training and works professionally as a tour guide. She was in Kyiv when the war started. She set off for Poland with her disabled mother and three cats on 25 February. At the start of the journey, she was not sure where they were going. She was also pregnant with her first baby. She had already been to Toruń several times, and she has good friends here; it was they who invited her to visit. They crossed the border on 26 February, by car. Most of her luggage were things for her cats and incontinence underwear for her mother.

Interviewee no. 3 is 40 years old. She is a historian and was in Kharkiv, Ukraine, on the first day of the war. For a long time she did not make the decision to leave, and when she finally did, she and her household were the last people to leave their flat. Between 5 and 6 March she packed her suitcases, and on 7 March she set off. She crossed the border on 8 March, by train. On 10 March she arrived in Toruń. She had never been to Poland before. A former student of hers lives in Toruń and was able to arrange a place for her to stay.

Although interviews were the main source of information for the project, legal regulations and official documentation produced by Polish and Ukrainian government institutions and international organizations, especially the UN Refugee Agency (UNHCR), were also analysed. The research also draws on reporting in the popular press, given the limited availability of relevant scholarly material at this time.

Before the war started, Magdalena Wiśniewska-Drewniak and James Lowry were awarded a grant to explore the Polish instances of displaced archives reported in the 2019/2020 ICA survey of archival claims. Given the circumstances, this project was postponed to respond to the more pressing questions raised in the Polish archival community related to the analogue and digital documentation of the war being carried into Poland by refugees. The data collected by Magdalena Wiśniewska-Drewniak and Nadiia Kravchenko has been analysed by Magdalena Wiśniewska-Drewniak and James Lowry in the following section.

### Preliminary findings: some directions for further work

#### “What I have with me”

Interviewees consistently reported collecting hardcopy documents relating to themselves and their families before fleeing, including identity documentation such as passports, birth certificates, diplomas and other proof of qualifications, and documentation related to property ownership.

Interviewee no. 2 explicitly said that she placed more importance on documents than other material things:

“I took all possible documents, most importantly, not things, but documents, that is, everything... For the flat, everything I own, all my diplomas, everything I could use. Because I understood that if something happened to the house, I would need these documents.”

This attention to documentation might be expected given the professional profile of the interviewees (two historians and a landscape designer), who are used to working with documentation. We cannot exclude the possibility that Ukrainian or post-Soviet cultural norms also informed the consistent recognition of the value of official documents. A fuller study with a broader demographic range and closer consideration of the particular documents selected by refugees may provide sociological insights into the documentary behaviours and expectations of refugees.

In none of the interviews was there any information about taking documents such as family photographs or anything of symbolic, emotional or historical value. The only value that the documents taken with them had was of practical and evidential nature. However, it should be noted that all interviewees either left the country in a hurry and/or with little luggage, which may have significantly influenced their choices in terms of what to take with them. Perhaps the decisions of those leaving the country after a longer period of planning would have been different.

Interviewee no. 1 said that in the case of the COVID vaccination certificate, which was still required when she crossed the border, she had it on the Ukrainian government-provided mobile phone app (Diia 2.0), but she also took the paper version with her. The digital documents available in Diia are equivalent to their hardcopy versions under Ukrainian regulation, so the paper certificate was simply a second version of the document, a back-up. It may be worth considering in the future how far people (and refugees in particular) are able to trust the digital version of a significant document in an emergency situation when two versions of information (digital and analogue) are available and equivalent, while the digital document is available in the cloud (which servers, in those extreme circumstances, may be physically damaged or hacked) and on a mobile phone (which may, for example, be discharged). Conversely, Nadiia Kravchenko has anecdotal evidence of Diia documentation being the only surviving source of some official information for Ukrainians whose physical records have been lost or destroyed.

The Diia mobile application mentioned by Interviewee no. 1 is an element of a greater platform of digital public services for Ukrainian citizens. Currently, the Diia (meaning ‘action’) portal has more than 3.6 million users and it provides over 50 different online services. Via the mobile application (Diia 2.0) users can access nine types of personal documents (ID card, foreign biometric passport, student card, driver’s licence, vehicle registration certificate, vehicle insurance policy, tax number, birth certificate, IDP certificate)—they have the same legal force as their hardcopy versions. Apart from that, a number of online public services are available via the app (not all, some numbers are still available only via a computer) (Ministry of Foreign Affairs of Ukraine and Ukrainian Institute n.d.). The rapid process of digitalisation of government services, which started ramping up in earnest in 2019, accelerated during the Covid-19 pandemic. Today, Ukraine is one of the leaders in digital government services for citizens (Matuszak 2021).

It seems that this suite of administrative public services available "in the pocket" of every citizen may be of significant importance for displaced persons, both internally and externally. The impact of mobile applications providing access to governmental documents, information and public services on the information behaviour of refugees could be another important direction for future research. As well as the associated inconveniences and risks—both to the security of individuals and their personal data, but also to the infrastructure itself, which can, after all, become the target of attacks—both digital and physical—in the face of war but also in the time of peace (Tett 2022; Satter and Pearson 2022).

#### (Not) recording the war

One of the aims of the project was to find out whether and to what extent people experiencing war and becoming refugees document these events, e.g. by taking photos or videos. With such a limited number and selection of interviewees, our findings do not allow generalisation, especially as recording what is happening around us can depend on many factors (specific situations, e.g. whether someone might get in trouble for documenting what they are experiencing; the ways in which a person uses a mobile phone; their nature and approach to recording anything and keeping these documents; technological issues, and therefore the person's equipment capabilities; events witnessed by the person).

None of the three interviewees consistently documented war-related events or their experiences (including the experience of fleeing the country), or did so to a very limited extent. Interviewee no. 1 explicitly stated that she did not document the events of the war because she had previously come across traumatising photographs on social media depicting dead children, among other things. As she herself is a young mother, these images affected her greatly:

Interviewer: Did you take any photos and videos and document the course of the war after the outbreak of the hot phase?

Interviewee: No, I didn't.

Interviewer: Perhaps some photos at the border, or already after you have moved?

Interviewee: I didn't take photos because... Now social media, and shots that were taken by other people, specifically of dead children, as I have a child myself... I closed it all down and I try not to go there, if possible, I don't want to see it…

It is worth noting the young (27) interviewee's perception of the question and identification of the purpose of taking photographs/videos with sharing them on social media. As a result of this young person's previous traumatisation by material present on social media, not only did she stop using it, but she did not take any photographs or videos herself—she had no intention in publishing them on social media, so she did not take them at all. Later, after settling in Poland, she documented her participation in supplying humanitarian aid to Ukraine, in order to confirm delivery for donors and organisers.

Whether or not there is a generational assumption that the purpose of taking photographs and videos is to share them on social media, there is an active online community of young Ukrainian people, including soldiers and refugees, using social media to document the war for information sharing, reporting and awareness raising, and morale boosting and entertainment, for example Ukrainian soldier Tik Tok or the posts by Valeria Shashenok, reported as “Ukrainian Woman Documents What Her Family Cooks and Eats Inside Bomb Shelter via Tik Tok” (2022). Given that technology access and proficiency can be informed by age and economic status, in the increasingly visual and technologically enabled documentary practices of today, there is a question for memory workers around whose experiences of the war are being preserved.

Interviewee no. 2 responded:

“We left in the morning of the 25th, and frankly, there was no time for photos. And there was nothing to photograph, just an empty city...”

It is worth noting that this woman was leaving her home during the most hectic start of the war (the day after the attack), moving with her disabled mother and pets. It is therefore not surprising, and will probably be true of many refugee experiences, that these are extreme and difficult situations and very often refugees are preoccupied with other more crucial and urgent issues and events, so recording the experience is not a priority. At the same time, Interviewee no. 1 feels that her experience of observing the empty city is not worth recording. This is of course a subjective feeling, especially given the affective potential of images depicting the unusual emptiness in the context of the ordinary (previous) activity/vitality of the city (the so-called 'meaningful absence') (Wolf 2019).

Interviewee no. 2 only took one video of an important event, that is, the dangerous situation of the downing of a drone in the immediate vicinity of the border crossing. At the same time, she notes that her friends with whom she travelled took other photographs, and she now regrets not doing so. She also mentions that her friends who picked her up on the Polish side of the border also took photographs. However, all this material causes trauma in the interviewee and she does not want to return to it now (but she suspects that perhaps she will use them in the future):

Interviewee: [...] Honestly, I can't look at these photos that we took, that my buddies took... At the border. I can't look at these videos either. And when we finally got to the Polish side, my friends who met me, they were taking photos... I can't look at them, honestly, it's hard for me, and I always just flip through those photos.

Interviewer: Do you intend to somehow... keep those materials as a memory for yourself, keep them somewhere?

Interviewee: I'm like, I do a lot of things like... keeping, because I am a historian, what I am is a guide, but... Maybe in a few years I'll be able to look at it. Let them lie there, but honestly... I've removed most of them, I've left a few because... I can't.

Interviewee no. 3, who left Ukraine the latest of all the women involved in the project, says she was in her flat for the vast majority of the time, and that nothing memorable happened, so she did not take any photographs. She did not record her journey to Poland either.

This information suggests that the ubiquity of mobile phones in the refugee population does not necessarily imply the wide-ranging visual documentation of the refugee experience. The preliminary interviews indicated that there is a tendency not to use photo and video functions to document the journey into Poland. Instead, the respondents only documented the more eventful moments, such as the shooting down of a drone over the border. It is the more extreme imagery that is shared within the Ukrainian community, but the preliminary study suggests that such images are kept for future reference but avoided in the present because of their potentially traumatic effects. This suggests that any research that centres the mobile phone, such as mobile phone ethnographies and particularly mobile phone visual ethnographies, must be developed through a trauma-informed research design process that would safeguard research participants.

#### Experiences at the border

The border between Poland and Ukraine is also the external border of the European Union and the Schengen Area—i.e. an area comprising 26 European countries within which there is no border control. Ukraine is not a member of either of these two areas (EU or Schengen), so the movement of people on this border is fully controlled and registered, and Polish border guards perform this control on behalf of all Schengen states (European Parliament and Council of the European Union 2016; European Commission—Migration and Home Affairs n.d.).

Before the war, the movement of people from Ukraine to Poland required the presentation of not only a passport, but also a Covid vaccination certificate, a negative Covid test or quarantine. Interviewee no. 1 crossed the border with her child under such rules (24 February 2022). A day later, a new “Ordinance of the Council of Ministers on the establishment of certain restrictions, orders and prohibitions in connection with the outbreak of an epidemic” was published (entered into force on 28 February), which added a provision to exclude from the restrictions related to entry into Poland from outside the Schengen area persons entering Poland from Ukraine in connection with an armed conflict on the territory of that country (Republic of Poland 2022a). Therefore, Interviewee no. 2 and no. 3 crossed the border into Poland under the new rules and the only document they had to present was their passport.

Interviewee no. 2, who crossed the border with her cats, also mentioned that she did not have to show her cats' passports when crossing the border, although she had the documents with her.

With such a massive refugee influx, the Polish authorities have implemented as simplified a border crossing procedure with Ukraine as possible, with a minimum number of documents required and minimal registration of the data of refugees. While this underscores the widely recognised contingency of official recordkeeping policies and practices, when read against reports of Nigerian, Indian and Egyptian refugees being delayed, sidelined or deprived by Ukrainian border guards at checkpoints en route to Poland and Hungary, apparently before any document checks to verify nationality took place, the experiences of our White interviewees raises questions about the racial aspects of these decisions on the ground (Busari et al. 2022). Although such an extreme situation must be complex, and the guards' desire to protect Ukrainian women and children may have come into play first, leaving foreign students (especially PoC, and therefore, at first glance, non-Ukrainians) in the back, nevertheless, it is important to note: Procedures may not articulate a racist exclusionary principle, but “there is an unofficial but equally real and consequential system of reading that is beyond the record as we have typically understood it”, with potential outcomes that can be damaging for anyone who is not White (Lowry 2022).

Regarding evidence of border crossings, the interviews consistently show that refugees did not obtain any documents from border guards and the only trace they have of their crossing the border was a stamp in their passports. Interviewee no. 2 mentions that she knows a story of a person who (probably due to a mistake) did not receive a stamp when crossing the border. As a result, this person could not apply for aid in Poland. This draws attention to the centrality and great relevance of this document to a refugee’s status and prospects for support.

#### Life in Poland: the PESEL number

Due to the fact that so little information about refugees is collected at the moment of crossing the border, it is only when the PESEL number is assigned that the Polish state registers the refugees' detailed personal data. PESEL (Powszechny Elektroniczny System Ewidencji Ludności—Universal Electronic Population Register System) is a register used to collect basic information on the identity and administrative and legal status of Polish citizens and foreigners residing in Poland. PESEL commonly refers to not so much a data set (currently managed by the Minister of Information Technology), but as the 11-digit numeric identifier itself.

Ukrainian citizens and their family members who arrived to Poland have the right to apply for a PESEL number, which gives them access to social benefits (e.g. social assistance and medical services), as well as the right to open a business or facilitate formalities with starting work in Poland (Republic of Poland 2022b). By 13 July 2022 over 1.220.000 citizens of Ukraine and their family members registered in the system and received their own PESEL number, more than 93% of whom were women or minors (UNHCR 2022).

All interviewees had experience of the process of applying for a PESEL number when they moved to Poland after the start of the war. Interviewee no. 1 had already had her PESEL before, but after crossing the border in February, she applied for a PESEL for her young child. All women mention that the procedure was standard and not very complicated. Problems during this process are only mentioned by Interviewee no. 2, whose mother is in a wheelchair. For legal reasons, the person applying for registration in the database has to appear at the office in person. Transporting a person in a wheelchair to the municipal office proved to be very challenging. In the end, transport was organised thanks to the kindness of one of the municipal clerks and her family who worked in the fire brigade.

People with disabilities are particularly vulnerable when they find themselves in a refugee situation. In May 2022, the Polish Commissioner for Human Rights pointed out several problems with assisting Ukrainian refugees with disabilities. He noticed that the organisation of assistance to war refugees with disabilities is based primarily on the activities of NGOs. This means that these activities are decentralised and require efficient coordination and precise information about forms of assistance that are effectively distributed among those in need. It is also difficult to apply for a disability certificate in Poland. Many disabled Ukrainian refugees have equivalent certificates issued by the Ukrainian government, but these are currently not recognised in Poland (Polish Commissioner for Human Rights 2022; Wójcik 2022).

### Other considerations for further research

Several other considerations for research emerge from this study, though not directly from our interview data: Rather, through the experience of being in Torun during our work together, our experiences of the research process, and our own encounters with our sources or lack of sources.

Abortion is illegal in Poland, and as of 1 October 2022, doctors will be required to report conceptions, which raises concerns about the goal of such registrations, including whether or not they will be used against women in the future, for example, to draw legal consequences for aborting a pregnancy abroad. With reports of women who have been sexually assaulted by Russian soldiers needing to reach Germany to receive appropriate medical care, the prospect of a conception register marks Poland as an increasingly hostile place to settle as a displaced person. Feminist networks have organised to support the movement of assault survivors across Poland and into other countries (Stefanek 2022). How can archival expertise be directed to support this work? What tools and techniques can be developed or re-engineered to reduce the risk of arrest and prosecution for rape survivors and the activists who are providing them care?

The cessation of construction work in Poland as a result of the exodus of Ukrainian workers, returning to defend Ukraine, raises questions about the digital parallel to Ukrainian return and repair. How is the Ukrainian diaspora channelling information and digital resources such as financial resources, practices of care, and the survival of lived Ukrainian culture, which Russia denies is distinct? Related to this notion of digital or informational resistance is the concept of informational warfare, or Virilio’s (2005) “information bomb”. The last decade has seen increased scholarship of informational warfare, responding to changing informational methods in conflict. Much of this research is cross-disciplinary, bringing technologists, social scientists and others into conversation, and arguably constituting an emergent field, signalled by the founding of the “Journal of Digital War” in 2020. Archival studies have made interventions in this discourse in relation to authenticity and disinformation in domestic spaces, but given Russian use of disinformation, surveillance and the targeting of information technologies and infrastructures today, an archival perspective on the international uses of weaponized information might provide valuable insights (for example, Brennan 2019).

As for analogue information and warfare, archival science has a long history of dealing with the uses and abuses of records in wartime and subsequently. Protections for cultural heritage during conflict and post-war repatriation have already been invoked in the local and international responses to the Russian invasion. The ongoing relevance of this work and the long term trajectory of battles over memory and evidence was vividly illustrated on the second day of the war, when Russian forces obliterated the archives of the Security Service in Chernihiv, which housed records of the Soviet repression of Ukrainians (INTERFAX – Ukraine 2022).

Apart from oral history projects organised by the academic community and the Aid Archives, we have not been able to access grassroots initiatives documenting the war in Ukraine and the refugee experience. However, this does not mean that such activities were not undertaken. One reason for this may be that activists, instead of opting for 'passive' aid (recording what is going on), are nevertheless moving towards active, humanitarian aid, which is more immediate and directly affecting people's lives. Perhaps we need to wait until the acute phase of the war is over for there to be initiatives focusing on collecting evidence of war and suffering—retrospectively; also in the context of rebuilding the state and local communities, looking for and judging the guilty, naming heroes, reckoning with the past and returning to normal life. Oral history has the potential to illuminate aspects of the refugee and diasporic experience. Future projects might be very valuable, both for the historical significance of such information, but also for the process of reconciliation or seeking justice. Yet, the traumatising potential of such projects (for both interviewees and interviewers) should be carefully considered.

Research in archival science can raise complex ethical and legal issues, particularly if it uses methods such as qualitative interviewing and deals with potentially sensitive issues. The NCU Faculty of History, where Magdalena Wiśniewska-Drewniak is employed, does not have an appropriate ethics committee and, at the same time, the opinions of such bodies in other departments or at a university-wide level cannot be drawn upon. This is an important argument in the discussion on the position of archival science vis-à-vis other scientific disciplines, in particular its traditional (still alive in Poland) attachment of archival science to history. It is worth mentioning that according to the list of disciplines of the Polish Ministry of Science and Higher Education, archivistics is not a separate scientific discipline (Republic of Poland 2018, and the project leader – Magdalena Wiśniewska-Drewniak – is formally assigned to the discipline of history, despite the fact that she has never conducted any research on the past. This suggests that work is needed to think through how ethics controls can be put in place for a field undergoing changes to its priorities and methods.

## Conclusion

Our research has sought to illuminate some of the issues that archival studies scholars, archivists, archival institutions and associations may consider as they attempt to work in solidarity with the Ukrainian people.

The preliminary data suggests that in exigency, Ukrainian refugees are likely to take significant documents with them, including paper copies of authoritative digital records. While e-government apps such as Ukraine’s Diia offer convenient access to a range of key official records, dependency on online access becomes more fraught in circumstances where aggressors are targeting both the servers that host the data via physical attacks and the integrity of the data itself through cyber attacks. On the other hand, Diia has provided access to information that has been lost or destroyed in its paper form. While web archiving is being used internationally to preserve the Ukrainian web presence, this public facing data is likely to be only a fraction of the digital information that is under threat, including personal data (records), the loss of which could greatly impact the lives of displaced people and their descendants. With initiatives such as SUCHO [n.d.] now focusing on distributing digitisation equipment to Ukrainian colleagues to help safeguard analogue information, and safe haven archives offering to house digital content that may not be in the public domain, there are questions to be considered about how individual refugees manage their digital records, many of them familiar to archivists: questions concerning originality, contextualising metadata, and the intersections of legal regimes and their attendant documentary requirements.

Only to a limited extent did our interviewees engage in the visual documentation of their experience, even from the relative safety of the border, and there was a tendency to receive, save and transmit images circulated by others, but chiefly for later reference. Whatever archival uses may later be made of these images—for war crimes prosecution, event reconstruction, commemoration and mourning—it is clear from our preliminary data that such visual records can be highly traumatic and have the potential to re-traumatise, and therefore such archival uses must be trauma-informed.

The limited visual documentation by our interviewees points to the possibility that the other affordances of the mobile phone may be more relevant to the production of documentation by refugees. It is likely that many meaningful documentary traces of the experience of flight are to be found in the text and voice messages through which refugees coordinated the evacuations of loved ones, shared information related to safe passage, wayfinding to the border, border crossing requirements and life over the border. Given the oral history projects already underway to understand the human experience of the war, it may be useful to refugees and researchers if these informal and ephemeral records were more widely recognised as sources worth preserving, but again, with highly traumatising potential.

Our findings, when read together with other research, including the work of defense analyst John Arquila and media researcher Roman Horbyk, suggest that Ukrainian civilians have participated in Ukraine’s tactical response to the invasion by using phones to transmit information for use in military manoeuvres, while the Ukrainian military has used them for “mapping minefields or wire-tapping the enemy or even targeting the artillery fire” (Stutchbury and Werden 2022; Stastna 2022). Civilian participation in Ukraine’s military response via mobile phones as information technologies, together with Russian signal jamming, hacking and disinformation campaigns—all experienced via the phone—evidence how information (technologies and practices) continue to become more granular and embedded, making it possible to speak of participatory warfare and the constitution of a digital frontline (Asmolov 2021).

The relative ease with which our interviewees crossed the Polish border provides a counterpoint to reports in the media regarding the exclusion of people of colour from border crossing queues by Ukrainian officials (Pronczuk and Maclean 2022). While the simplification of documentary requirements for immigration underscores the malleability of official policy when it comes to records, the racist targeting illuminates that official requirements are only one factor in the experience of rights and safety at the border: that outside of “the record” there are cultural and personal biases that are as powerful as records in their ability to exclude people from their rights and from safety. Furthermore, our data reveals that imperfect record practices, such as failure to stamp a passport, has further consequences—that each record participates in a chain of records, rights and entitlements that cannot always be anticipated but are often difficult to redress when something in that chain is anomalous or mishandled.

Beyond the border, access to services in Poland centres on the assignment of a PESEL number, and while this e-government system is advanced and is generally considered to be well-functioning, our interview data indicated that barriers to accessing a PESEL such as the requirement to be physically present at a local government office, could and did impose additional impediments to disabled people. Similarly, Ukrainian documentation of disability was not accepted as proof of disability under Polish regulations, (re)placing a burden of proof on disabled people. It is conceivable that benchmarks for proving disability may require medical documentation left behind in Ukraine, creating the possibility that a slate of medical tests may stand between the disabled person and access to social services. Taken together, the bar for records requirements appears to be set higher for disabled refugees than for others.

Although our study was seriously limited by the circumstances in which we were working and the availability of participants, the findings flag some key issues that the international archival community might consider as it seeks to support the Ukrainian people and their archives. More generally, even as our participants were very similar in their positionalities, the information they shared helped to surface some of the ways that state practices and records systems cause refugees to experience additional burdens or harms if they are women, disabled or people of colour. An awareness of this will be invaluable in designing records systems and processes that help to better create safety for those who have experienced warfare and displacement.

Russia’s ongoing violence in Ukraine continues to be condemned by much of the international community, including the international archival community, which has censured state archival institutions and excluded Russian archives and archivists from various forums. At the same time, China’s opposition to the ICA’s decision to break ties with Russian archives (ICA. Executive Board (2022) reminds us that archival solidarity is a staging ground for broader geopolitical positions and agendas. At this high level, it can be easy to lose sight of the situation for those on the ground. Listening to the experiences of Ukrainian refugees is one way to centre people as we design records systems and processes in our own countries and participate in archival solidarity internationally. The on the ground realities were recalled to us several times during this work. One of those moments occurred when a contact in Ukraine suddenly went silent: Through the Erasmus + programme, which uses funding from the European Union to support foreign exchange students, Magdalena Wiśniewska-Drewniak sought to fund five Ukrainian students to study in Poland. After no response from a Dean at the Ukrainian university, she enquired from colleagues and learned that despite his academic exemption, he had chosen to join the Territorial Defense Force and was engaged in armed combat.^4^ It is striking to think that as a Dean at Magdalena Wiśniewska-Drewniak’s University was welcoming James Lowry and Nadiia Kravchenko to the university, over the border his counterpart in Ukraine was in a Kevlar vest, with a gun in his hands, no doubt alongside some of his colleagues and students.

## Data Availability

The datasets generated and analysed during the current study (interviews’ audio recordings and their transcriptions) are not publicly available due to their sensitivity, in order to protect the interests of the participants.

## Footnotes

1 Only countries with more than 50.000 Ukrainian refugees were listed.

2 ‘Ukraińskie ślady na ziemi piotrkowskiej. Dokumenty z zasobu Archiwum Państwowego w Piotrkowie Trybunalskim’ / ‘Укpaїнcькi cлiди нa пйoтpкoвcькiй зeмлi Дoкyмeнти Дepжaвнoгo apxiвy в Пйoтpкoвi Tpибyнaльcькoмy’.

3 In Polish: „Dzisiejsza solidarność Polaków z Ukraińcami ukształtuje nasze wzajemne relacje na dziesięciolecia. To fundamentalne wydarzenie w naszej wspólnej historii. Nie pozwólmy, by pozostało nieudokumentowane”.

4 Arrangements have now been made for the students to go to Poland.
